# Supplementary material for: Onset of double subduction controls plate motion reorganisation
Source: Nat Commun. 2024 Feb 19;15:1513. doi: 10.1038/s41467-024-44764-8 (PMC10876953; doi:10.1038/s41467-024-44764-8)
Supplement: Supplementary file 1 — Supplementary Information [file 41467_2024_44764_MOESM1_ESM.pdf]

Supplementary Information for

Onset of Double Subduction Controls Plate Motion Reorganisation

Kuidi Zhang[1], Jie Liao[1,2,3]✉, Taras Gerya[4]

[1]School of Earth Sciences and Engineering, Sun Yat-Sen University, Guangzhou, China.

[2]Southern Marine Science and Engineering Guangdong Laboratory (Zhuhai), Zhuhai, China.

[3]Guangdong Provincial Key Lab of Geodynamics and Geohazards, Guangzhou, China.

[4]Department of Earth Sciences, Swiss Federal Institute of Technology Zurich, Zurich, Switzerland.

✉ e-mail: liaojie5@mail.sysu.edu.cn

Contents

Supplementary Fig. 1 Model setup 3

Supplementary Fig. 2 Model (C844L1T5) results 4

Supplementary Fig. 3 Model (C871L1T5) results 5

Supplementary Fig. 4 Supplement to Fig. 3a (velocity field) 6

Supplementary Fig. 5 Supplement to Fig. 3a (stress field) 7

Supplementary Fig. 6 Supplement to Fig. 3b (velocity field) 8

Supplementary Fig. 7 Supplement to Fig. 3b (stress field) 9

Supplementary Fig. 8 Supplement to Fig. 3c (velocity field) 10

Supplementary Fig. 9 Supplement to Fig. 3c (stress field) 11

Supplementary Fig. 10 Supplement to Fig. 3d (velocity field) 12

Supplementary Fig. 11 Supplement to Fig. 3d (stress field) 13

Supplementary Fig. 12 Supplement to Fig. 3e (velocity field) 14

Supplementary Fig. 13 Supplement to Fig. 3e (stress field) 15

Supplementary Fig. 14 Models results testing resolution 16

Supplementary Table 1 Physical properties 17

Supplementary Reference 17

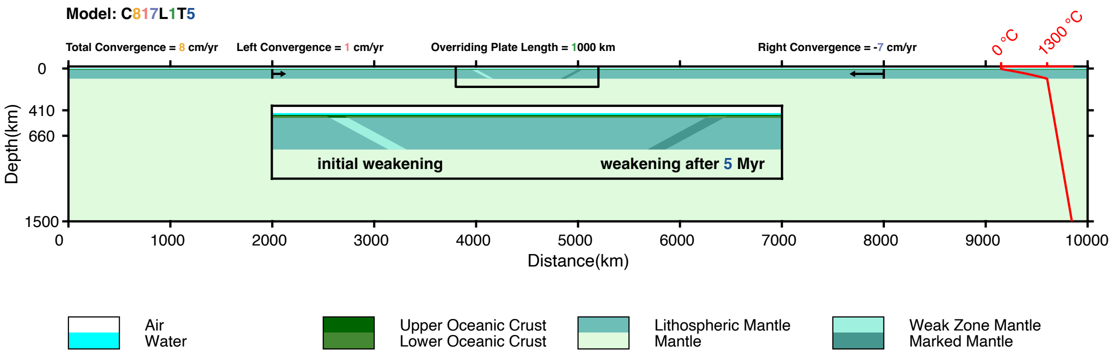

Supplementary Fig. 1 Model setup. The red line shows the geothermal gradient. Weak zone induced older subduction and marked mantle will weaken after 5 Myr in the model to induce the second subduction.

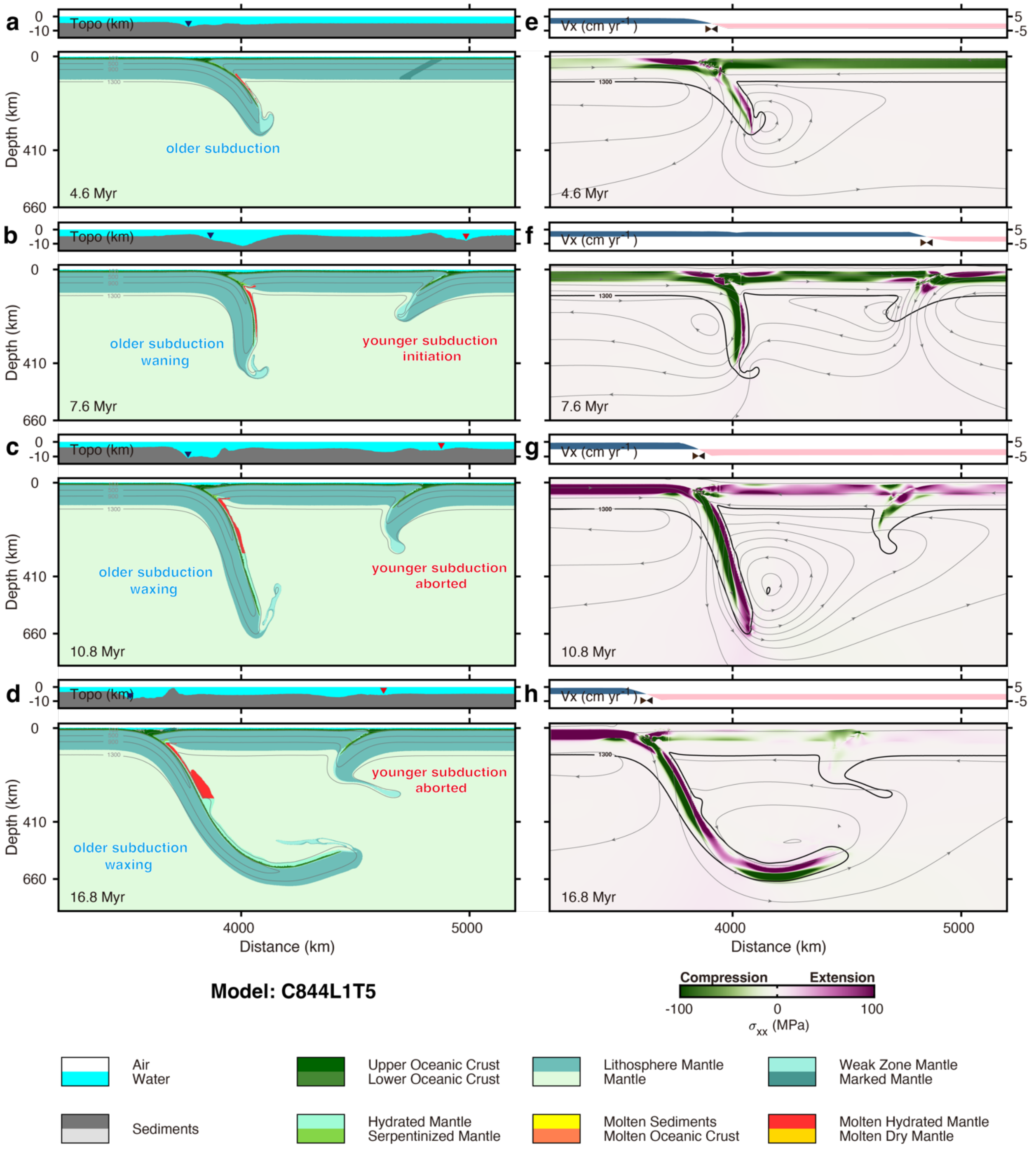

Supplementary Fig. 2 Model C844L1T5 (labelling logic see Fig. 2 in the main text) results showing the dynamic evolution of younger subduction initiation induced but aborted. a-d, Top: topography snapshots with dark blue and red triangles marking the trenches of the older and younger subductions, respectively; bottom: lithology snapshots with temperature contours (gray lines). e-h, Top: averaged horizontal velocity of the plates reflecting the main regions (double black triangles) absorbing convergence; bottom: stress evolution with temperature contours outlining the slab (1,300 °C, thick black lines) and the contours of stream function (thin gray lines with arrowheads) showing plate motion and mantle convection.

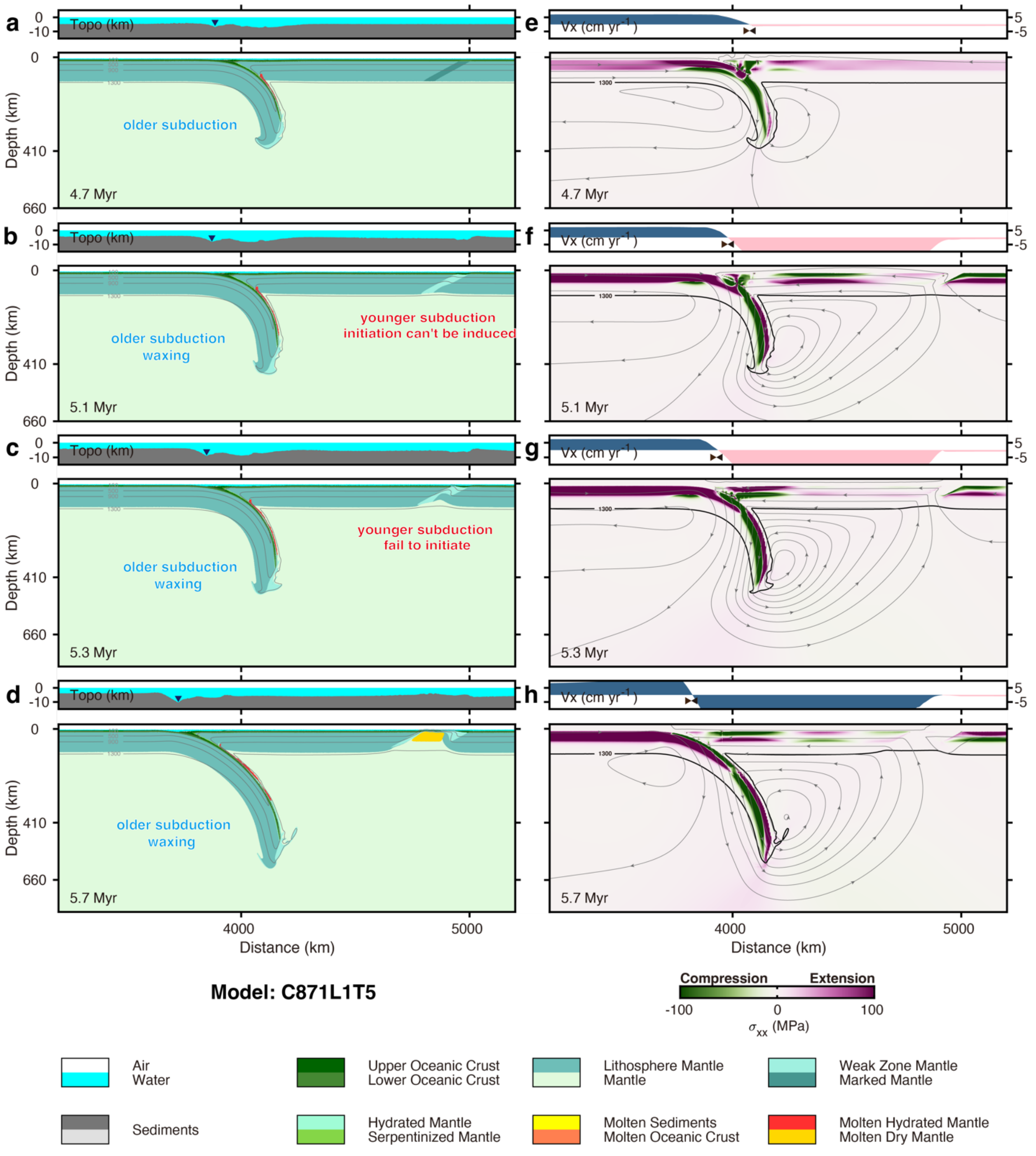

Supplementary Fig. 3 Model C871L1T5 (labelling logic see Fig. 2 in the main text) results showing the dynamic evolution of younger subduction initiation can't be induced. a-d, Top: topography snapshots with dark blue marking the trench of the older subduction; bottom: lithology snapshots with temperature contours (gray lines). e-h, Top: averaged horizontal velocity of the plates reflecting the main regions (double black triangles) absorbing convergence; bottom: stress evolution with temperature contours outlining the slab (1,300 °C, thick black lines) and the contours of stream function (thin gray lines with arrowheads) showing plate motion and mantle convection.

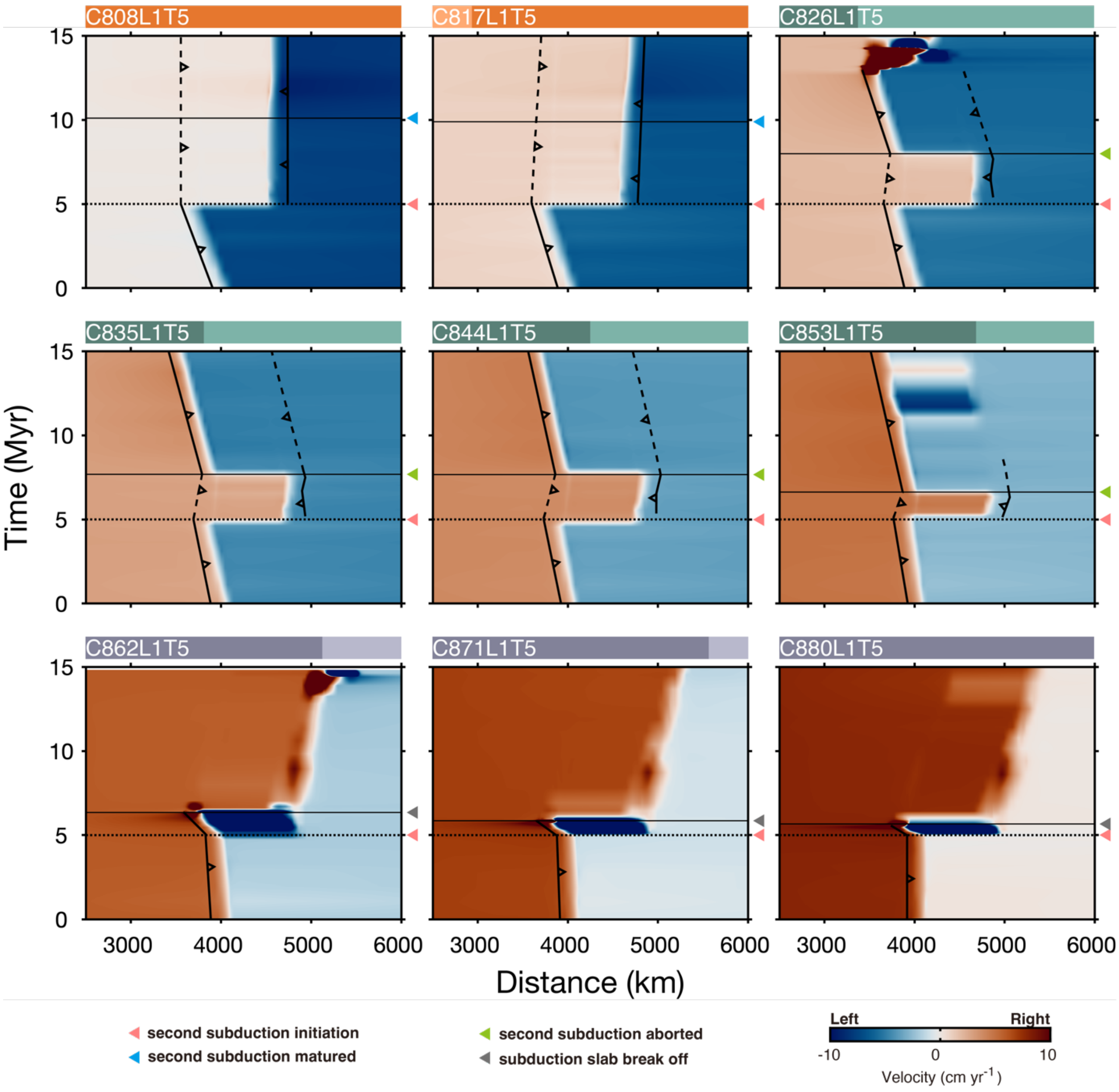

Supplementary Fig. 4 Model results testing plate convergence partitioning with overriding plate length of 1,000 km shown by plate velocity field (vertical lithospheric mean value; a supplement to Fig. 3a in the main text).

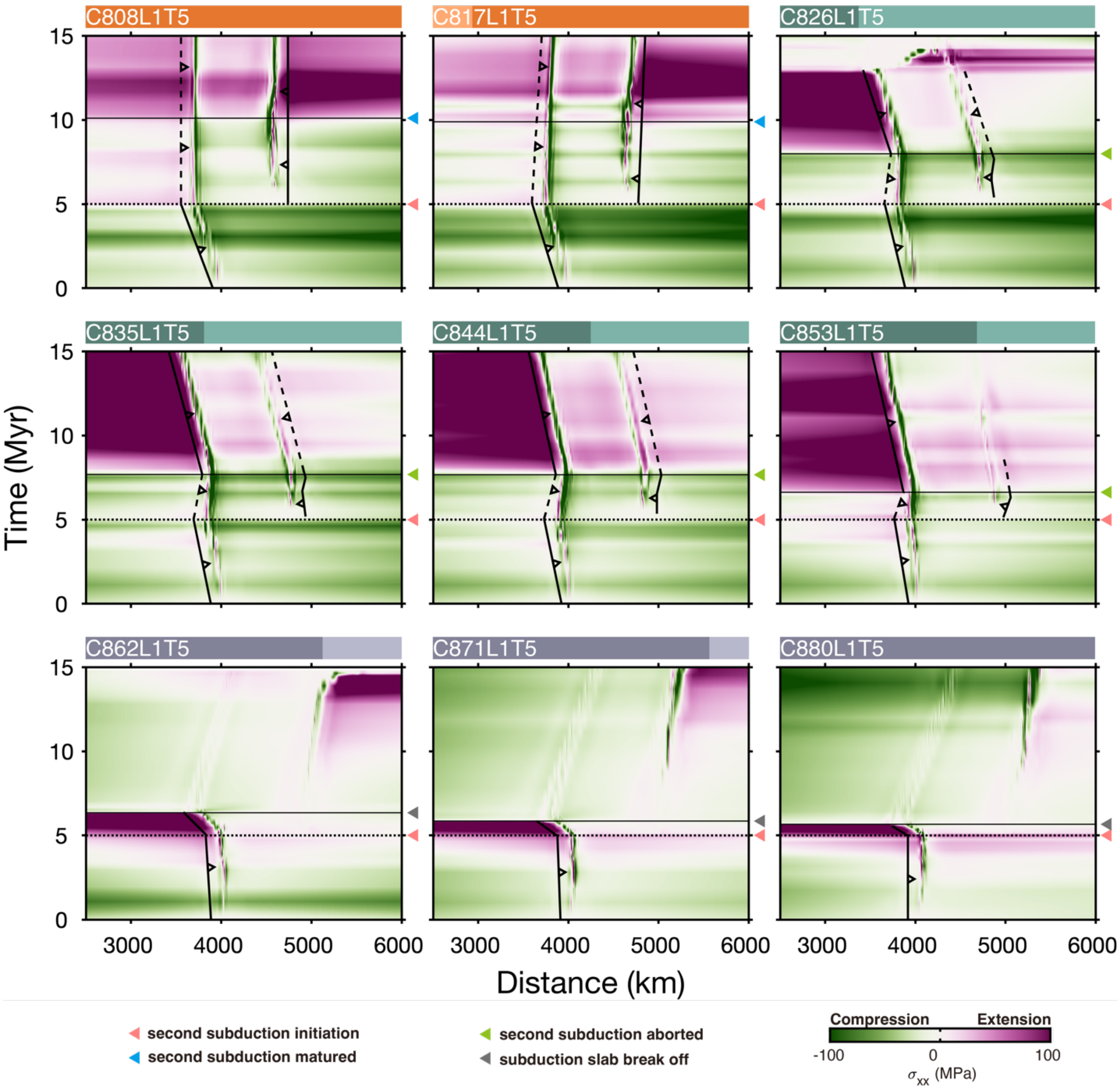

Supplementary Fig. 5 Model results testing plate convergence partitioning with overriding plate length of 1,000 km shown by horizontal stress field (vertical lithospheric mean value; a supplement to Fig. 3a in the main text).

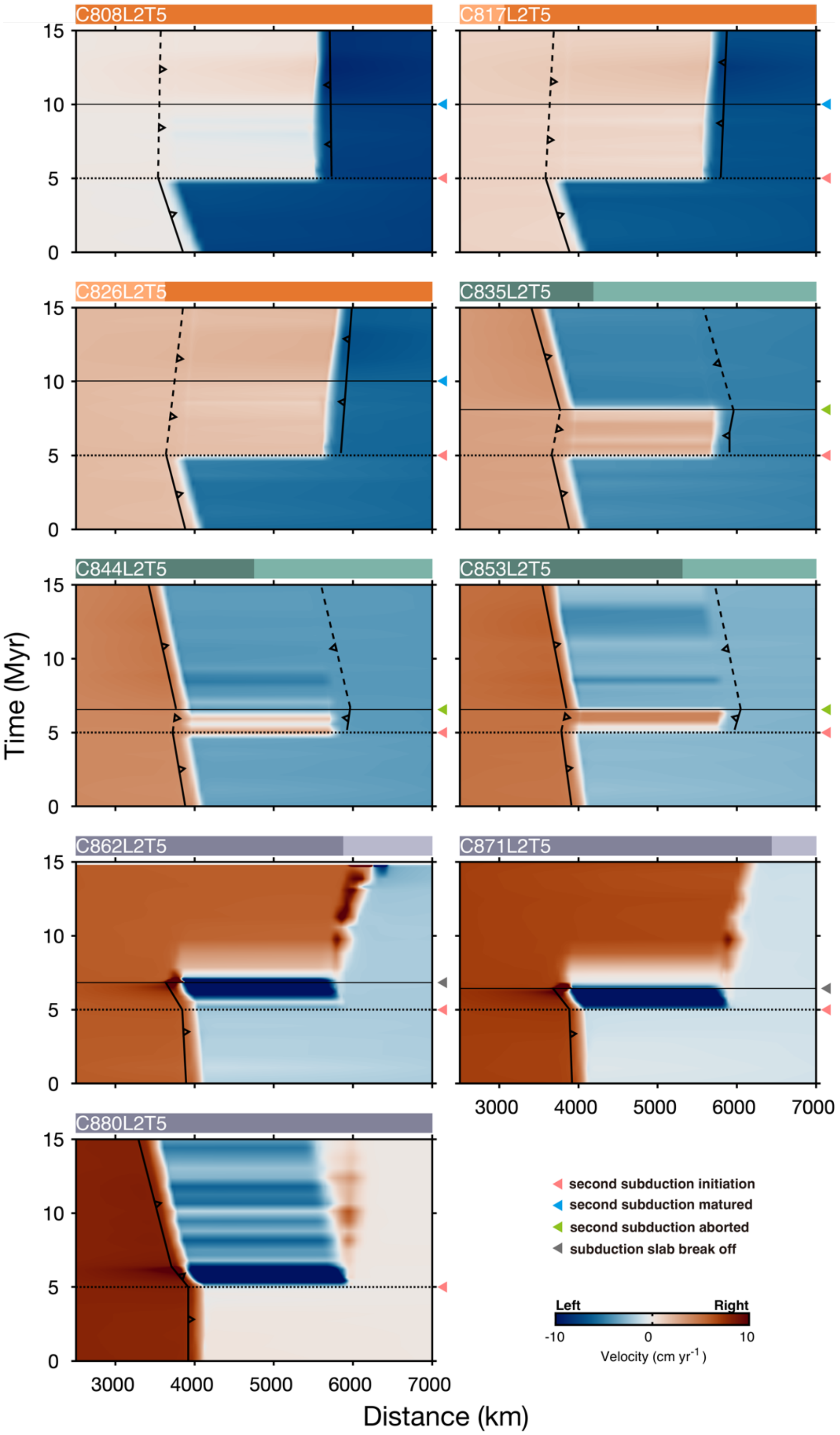

Supplementary Fig. 6 Model results testing plate convergence partitioning with overriding plate length of 2,000 km shown by plate velocity field (vertical lithospheric mean value; a supplement to Fig. 3b in the main text).

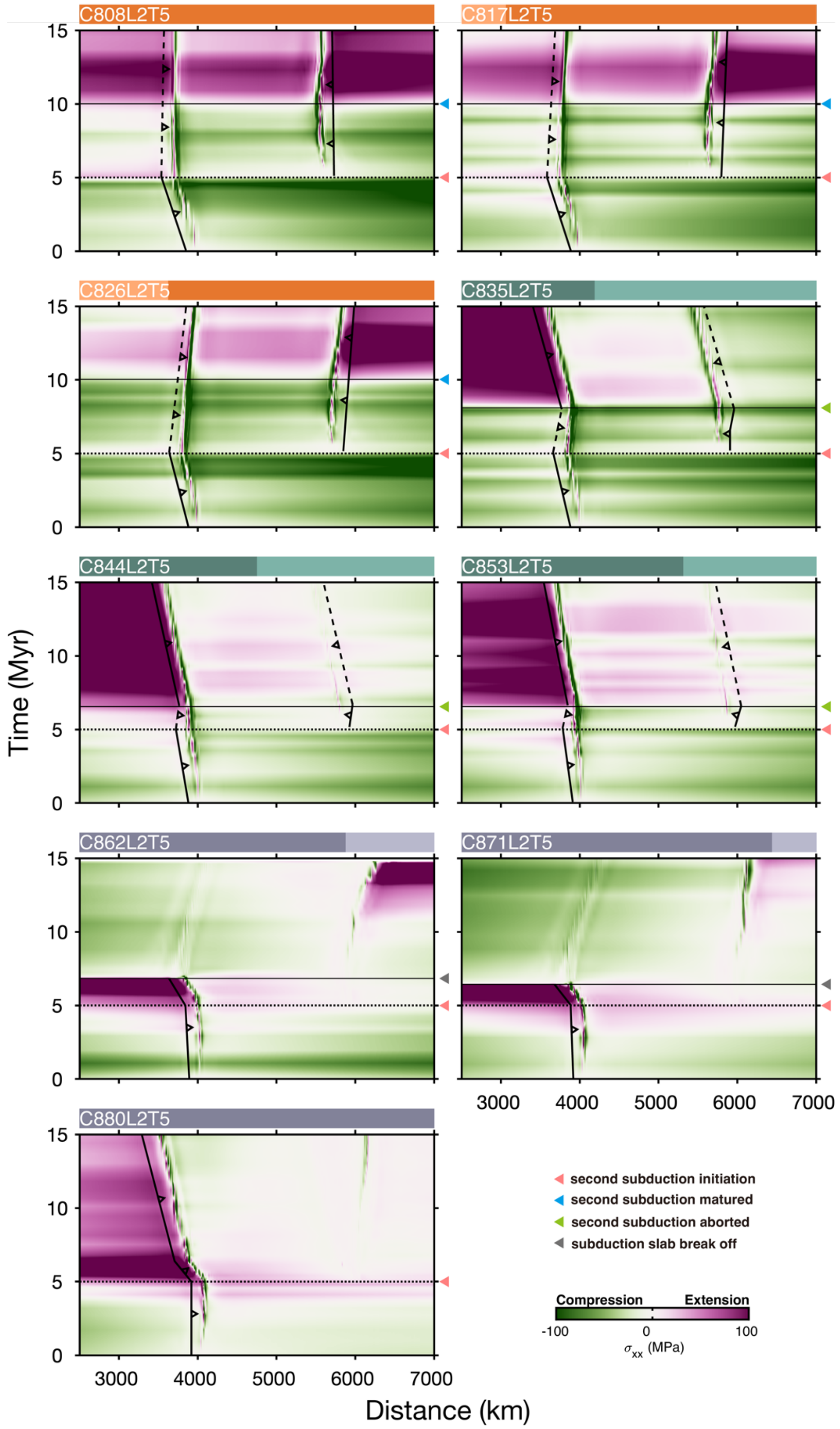

Supplementary Fig. 7 Model results testing plate convergence partitioning with overriding plate length of 2,000 km shown by horizontal stress field (vertical lithospheric mean value; a supplement to Fig. 3b in the main text).

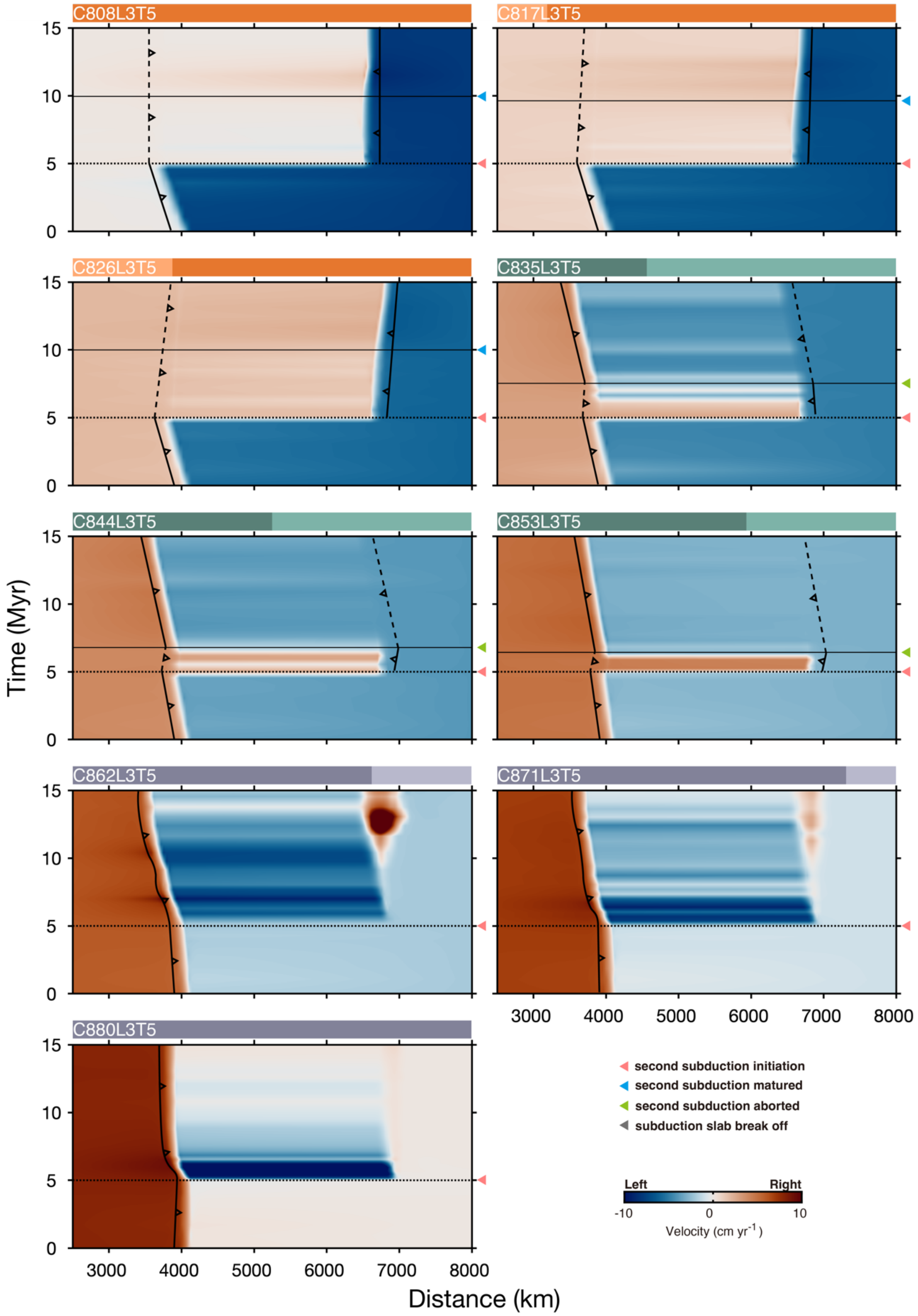

Supplementary Fig. 8 Model results testing plate convergence partitioning with overriding plate length of 3,000 km shown by plate velocity field (vertical lithospheric mean value; a supplement to Fig. 3c in the main text).

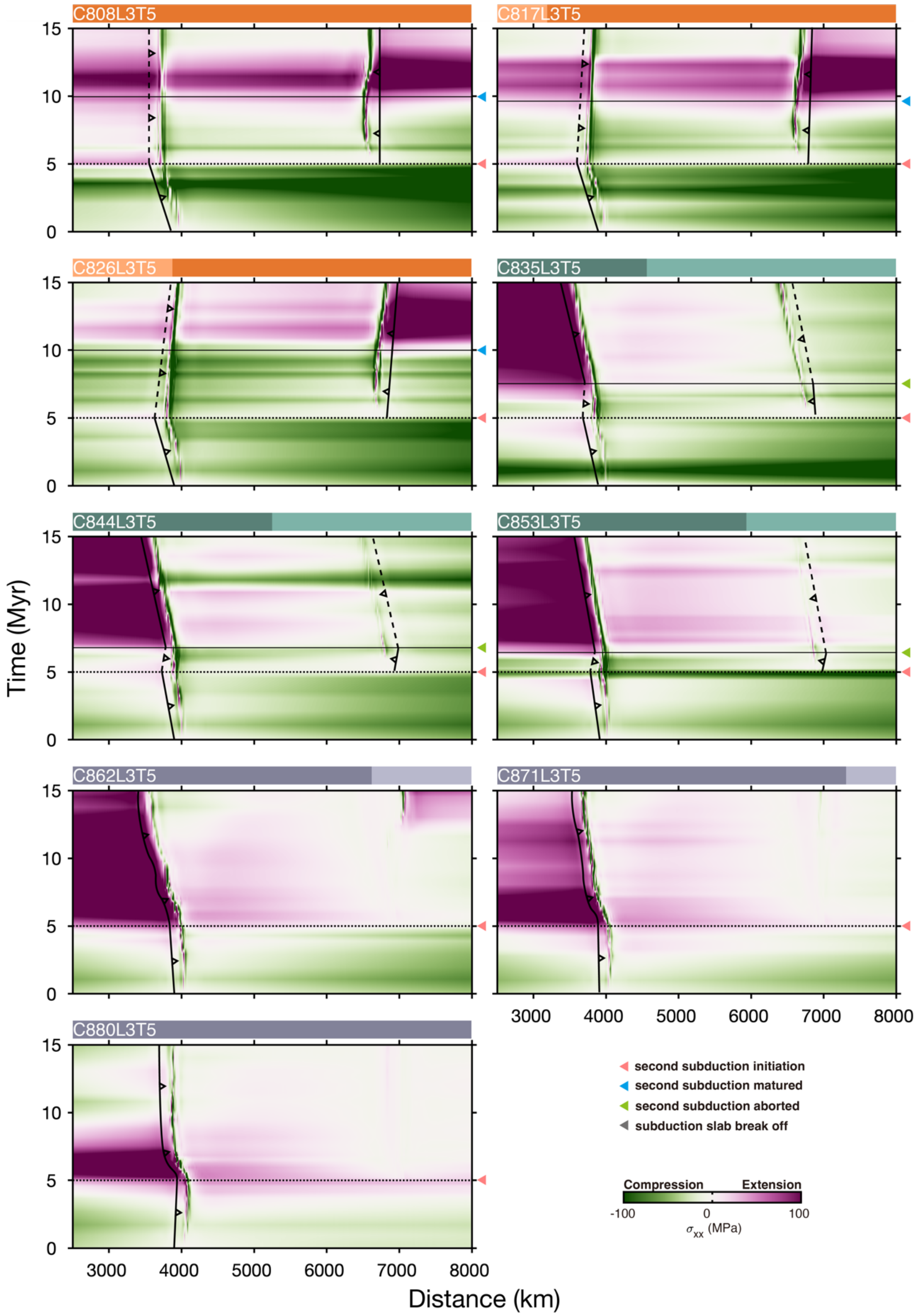

Supplementary Fig. 9 Model results testing plate convergence partitioning with overriding plate length of 3,000 km shown by horizontal stress field (vertical lithospheric mean value; a supplement to Fig. 3c in the main text).

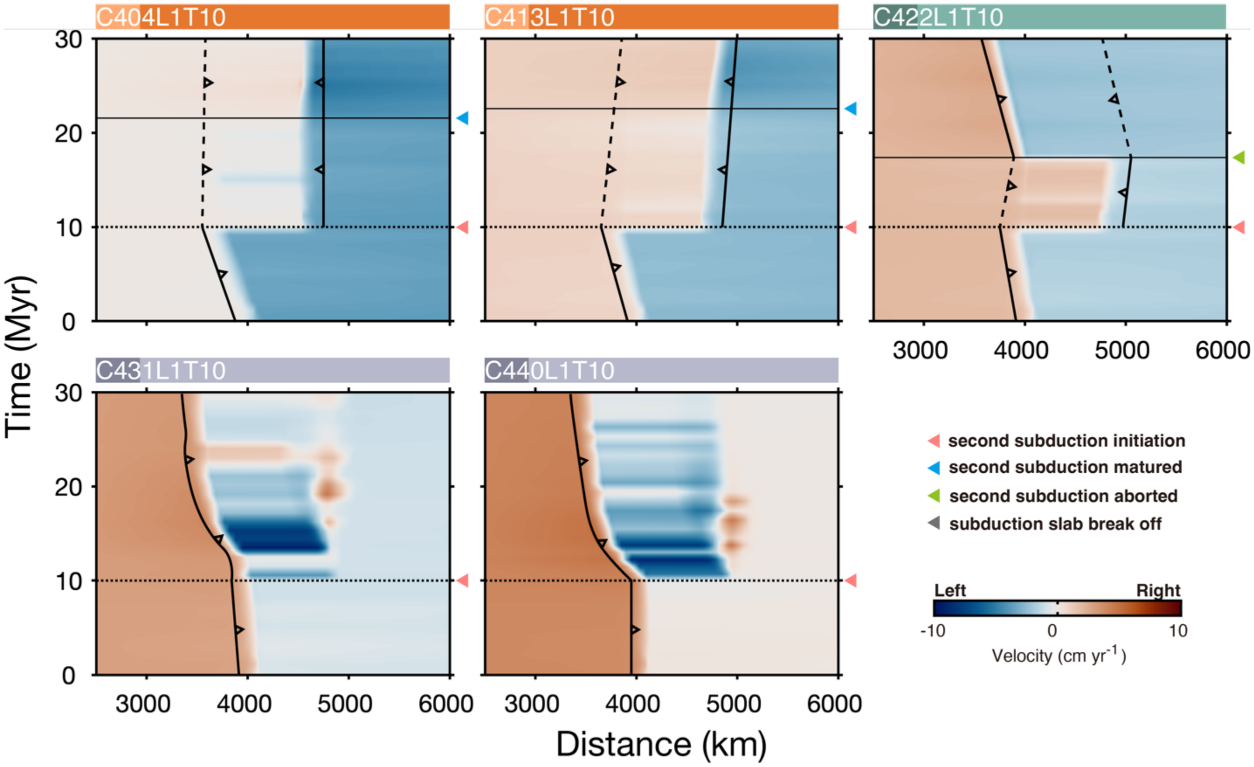

Supplementary Fig. 10 Model results testing plate convergence partitioning total convergence rate of 4 cm yr^{-1} shown by plate velocity field (vertical lithospheric mean value; a supplement to Fig. 3d in the main text).

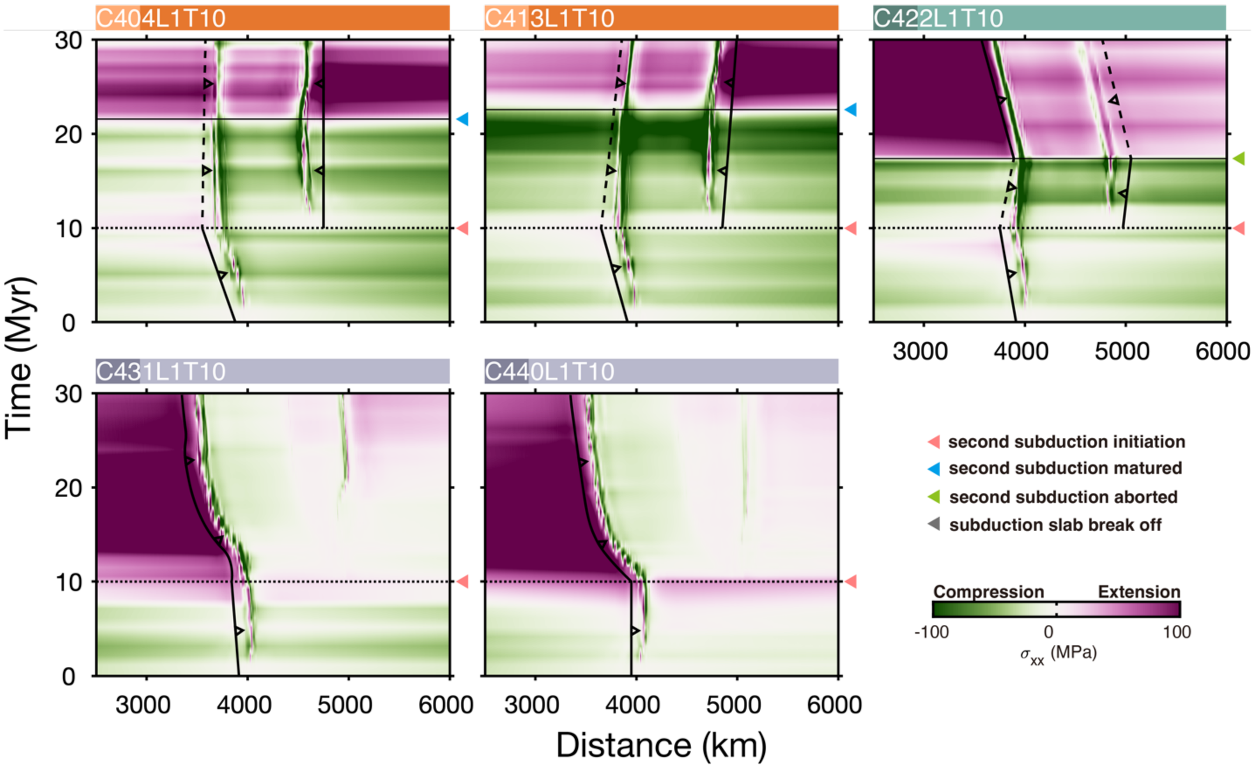

Supplementary Fig. 11 Model results testing plate convergence partitioning total convergence rate of 4 cm yr^{-1} shown by horizontal stress field (vertical lithospheric mean value; a supplement to Fig. 3d in the main text).

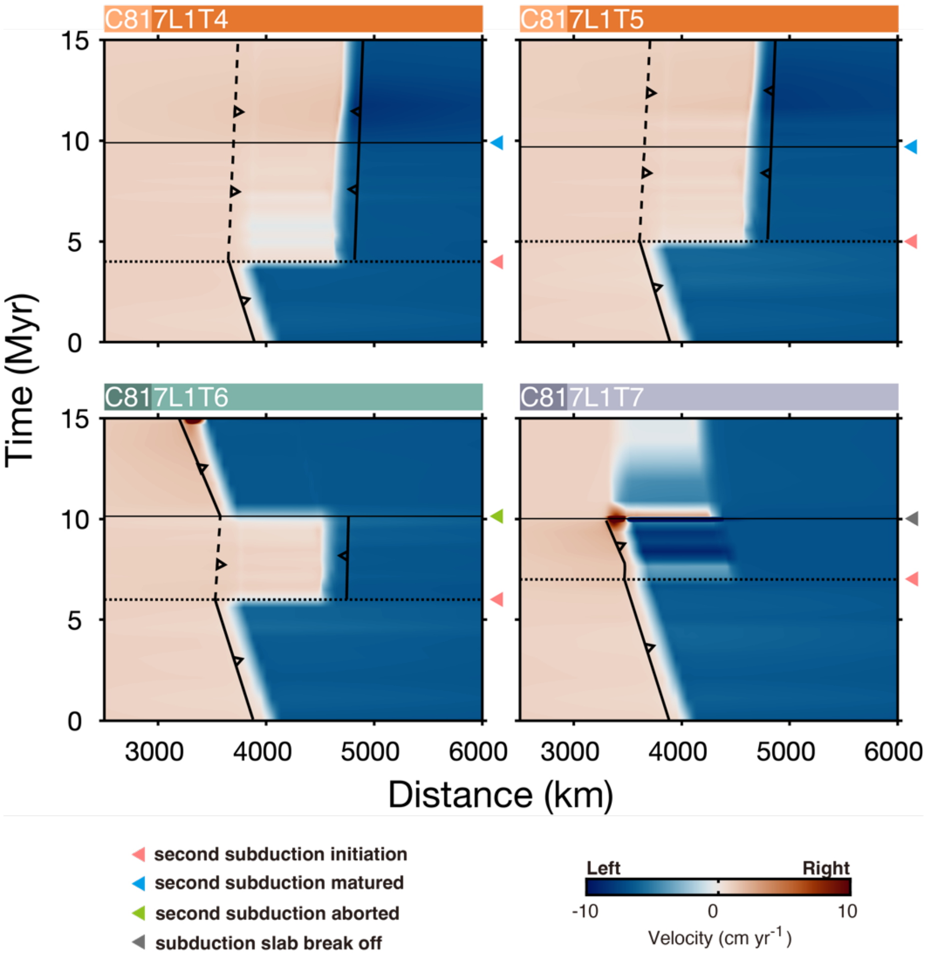

Supplementary Fig. 12 Model results testing onset time of younger subduction shown by plate velocity field (vertical lithospheric mean value; a supplement to Fig. 3e in the main text).

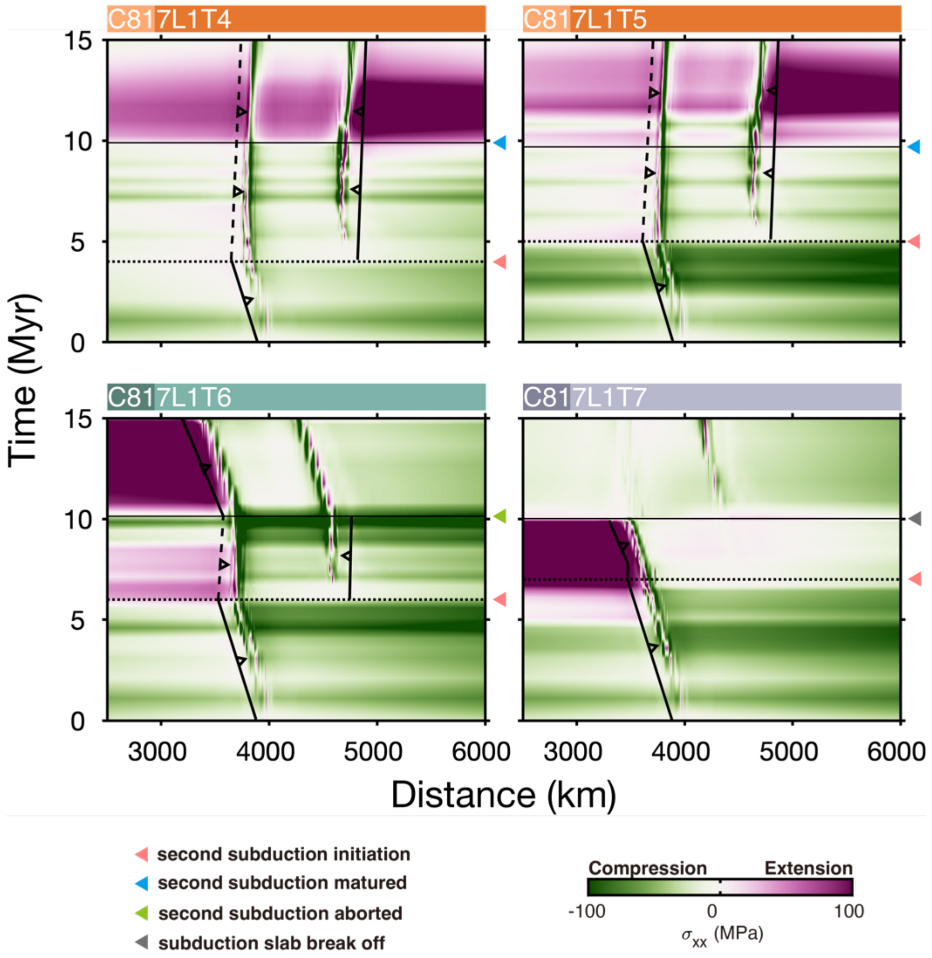

Supplementary Fig. 13 Model results testing onset time of younger subduction shown by horizontal stress field (vertical lithospheric mean value; a supplement to Fig. 3e in the main text).

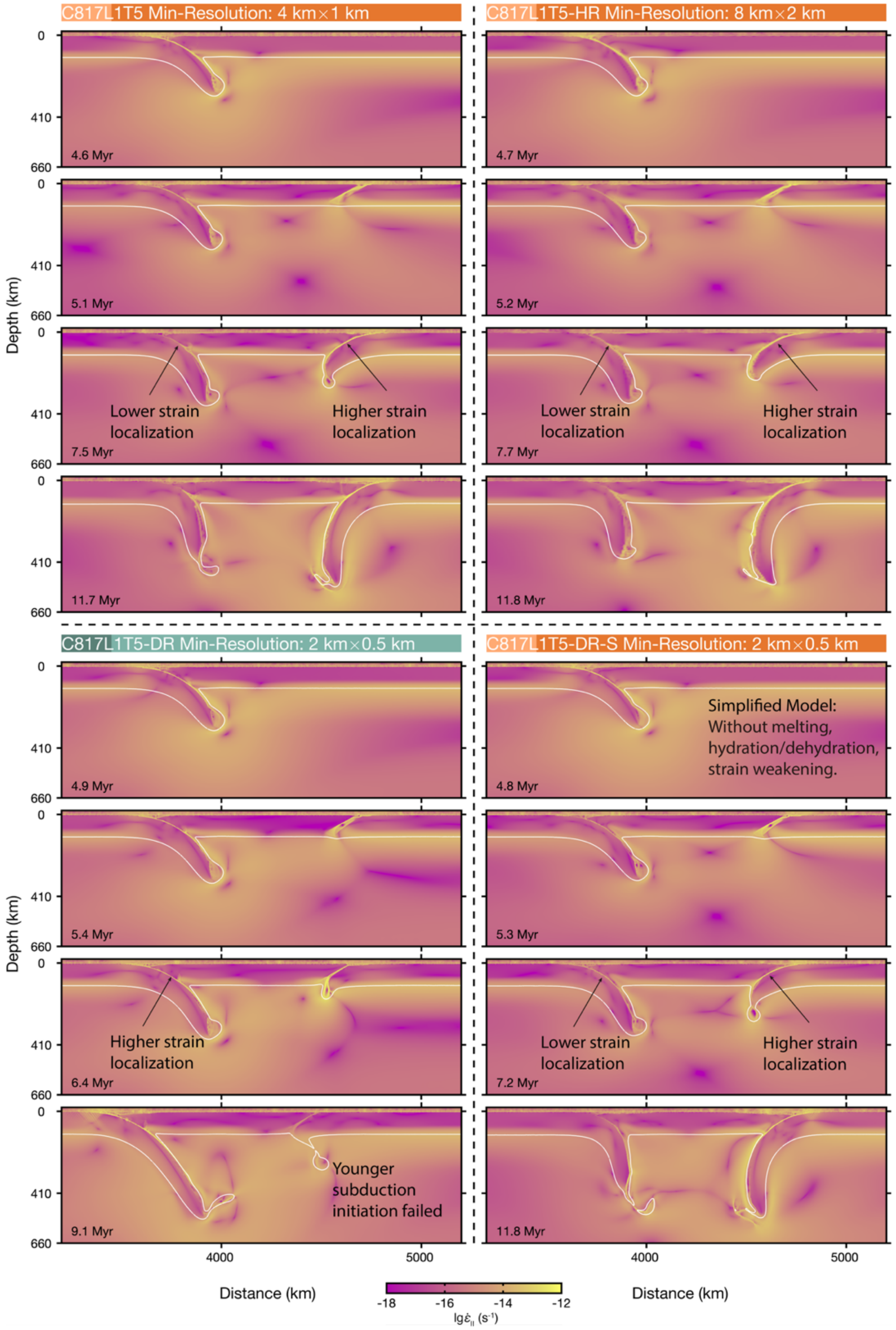

Supplementary Fig. 14 Model results testing resolution shown by strain rate ($\dot{\varepsilon}_{II}$) field. Model C817L1T5 is the reference model. Model C817L1T5-HR with half resolution: the younger subduction initiates and model results maintain the same as before; Mode C817L1T5-DR with double resolution: the younger subduction fails to initiate; Model C817L1T5-DR-S with double resolution but using simple petrological model (by neglecting melting and hydration/dehydration) without strain weakening: the younger subduction initiates as that in the reference model (more details see the main text in the "Method-Resolution Test section"). Temperature contours outlining the slab (1,300 °C, white line).

Supplementary Table 1 Physical properties

**Material**	$\boldsymbol{\rho_0}$[1, 2] (kg m^{-3})	$\boldsymbol{k}$[3, 4] (W m^{-1} K^{-1}) T (K), P (MPa)	$\boldsymbol{T_{\text{solidus}}}$[5, 6] T (K), P (MPa)	$\boldsymbol{T_{\text{liquidus}}}$[5, 6] T (K), P (MPa)	$\boldsymbol{H_{\text{r}}}$[1] (μW m^{-3})	$\boldsymbol{H_{\text{L}}}$[1, 2] (kJ kg^{-1})	**Flow law**[7]
Sediment	2,600 (solid)<br>2,400 (molten)	$(0.64 + \frac{807}{T+77}) \cdot \exp(4 \times 10^{-5} \cdot P)$	$\begin{cases} 889 - \frac{18{,}900}{(P+54)} + \frac{20{,}200}{(P+54)^2}, & P \leq 1{,}200 \\ 831 + 0.06 \cdot P, & P > 1{,}200 \end{cases}$	$1{,}262 + 0.009 \cdot P$	2	300	Wet quartzite<br>$c = 1$ MPa<br>$\mu = 0$
Basalt	3,000 (solid)<br>2,900 (molten)	$(1.18 + \frac{474}{T+77}) \cdot \exp(4 \times 10^{-5} \cdot P)$	$\begin{cases} 973 - \frac{70{,}400}{(P+354)} + \frac{77{,}800{,}000}{(P+354)^2}, & P \leq 1{,}600 \\ 935 + 0.0035 \cdot P + 0.0000062 \cdot P^2, & P > 1{,}600 \end{cases}$	$1{,}423 + 0.105 \cdot P$	0.250	380	Wet quartzite<br>$c = 1$ MPa<br>$\mu = 0.01$
Gabbro							Plagioclase An_{75}<br>$c = 10$ MPa<br>$\mu = 0.6$
Lithosphere-asthenosphere mantle	3,300 (solid)<br>2,900 (molten)	$(0.73 + \frac{1293}{T+77}) \cdot \exp(4 \times 10^{-5} \cdot P)$	P-H$_2$0-dependent		0.022	400	Dry olivine<br>$c = 20$ MPa<br>$\mu = 0.6$
Weak zone	3,300						Wet olivine<br>$c = 1$ MPa<br>$\mu = 0$
Hydrated mantle/<br>Serpentinized mantle	3,250/<br>3,250						Wet olivine<br>(strain weakening)<br>$c_0 = 10$ MPa<br>$c_1 = 1$ MPa<br>$\mu_0 = 0.1, \mu_1 = 0.05$

Flow law[7]:
Wet quartzite: $A = 1.97 \times 10^{17}$ Pa^n s, $n = 2.3$, $E = 154$ kJ mol^{-1}, $V = 8, 3.2$(serpentinized) J mol^{-1} MPa^{-1}.
Plagioclase An_{75}: $A = 4.80 \times 10^{22}$ Pa^n s, $n = 3.2$, $E = 238$ kJ mol^{-1}, $V = 8$ J mol^{-1} MPa^{-1}.
Dry olivine: $A = 3.98 \times 10^{16}$ Pa^n s, $n = 3.5$, $E = 532$ kJ mol^{-1}, $V = 10$ J mol^{-1} MPa^{-1}.
Wet olivine : $A = 5.01 \times 10^{20}$ Pa^n s, $n = 4.0$, $E = 470$ kJ mol^{-1}, $V = 8$ J mol^{-1} MPa^{-1}.

Supplementary References

1. Turcotte DL, Schubert G. *Geodynamics*. Cambridge university press (2002).

2. Bittner D, Schmeling H. Numerical Modelling of Melting Processes and Induced Diapirism In the Lower Crust. *Geophysical Journal International* **123**, 59-70 (1995).

3. Clauser C, Huenges E. Thermal conductivity of rocks and minerals. *Rock physics and phase relations: a handbook of physical constants* **3**, 105-126 (1995).

4. Hofmeister AM. Mantle values of thermal conductivity and the geotherm from phonon lifetimes. *Science* **283**, 1699-1706 (1999).

5. Schmidt MW, Poli S. Experimentally based water budgets for dehydrating slabs and consequences for arc magma generation. *Earth and Planetary Science Letters* **163**, 361-379 (1998).

6. Katz RF, Spiegelman M, Langmuir CH. A new parameterization of hydrous mantle melting. *Geochem Geophy Geosy* **4**, (2003).

7. Ranalli G. *Rheology of the Earth*. Springer Science & Business Media (1995).
